# Selenoprotein P deletion ameliorates metabolic stress-associated anxiety-like behavior in male mice

**DOI:** 10.1210/endocr/bqag010

**Published:** 2026-01-30

**Authors:** Guzel Gafiyatullina, Anna Shabalova, Hisanori Goto, Hein Ko Oo, Kengo Saito, Ryota Tanida, Qifang Li, Kyoko Kamoshita, Cynthia M Galicia-Medina, Yujiro Nakano, Yumie Takeshita, Kiyo-Aki Ishii, Hiroaki Takayama, Chiharu Tsuji, Haruhiro Higashida, Yohei Shinmyo, Hiroshi Kawasaki, Hiromasa Tsujiguchi, Akinori Hara, Hiroyuki Nakamura, Toshinari Takamura

**Affiliations:** Department of Endocrinology and Metabolism, Kanazawa University Graduate School of Advanced Preventive Medical Sciences, Ishikawa 920-8640, Japan; Division of Basic Science and Translational Research, Kanazawa University, Kanazawa 920-8640, Japan; Non-coding RNAs and RNA-Based Therapeutics, Italian Institute of Technology, Genova, Liguria 16163, Italy; Department of Endocrinology and Metabolism, Kanazawa University Graduate School of Advanced Preventive Medical Sciences, Ishikawa 920-8640, Japan; Department of Endocrinology and Metabolism, Kanazawa University Graduate School of Medical Sciences, Ishikawa 920-8640, Japan; Department of Endocrinology and Metabolism, Kanazawa University Graduate School of Medical Sciences, Ishikawa 920-8640, Japan; Department of Medical Neuroscience, Kanazawa University Graduate School of Medical Sciences, Ishikawa 920-8640, Japan; Department of Endocrinology and Metabolism, Kanazawa University Graduate School of Medical Sciences, Ishikawa 920-8640, Japan; Department of Biochemistry and Molecular Biology and Oncology, Cumming School of Medicine, University of Calgary, AB T2N 4N1, Calgary, Canada; Department of Endocrinology and Metabolism, Kanazawa University Graduate School of Medical Sciences, Ishikawa 920-8640, Japan; Department of Endocrinology and Metabolism, Kanazawa University Graduate School of Medical Sciences, Ishikawa 920-8640, Japan; Department of Endocrinology and Metabolism, Kanazawa University Graduate School of Medical Sciences, Ishikawa 920-8640, Japan; Department of Endocrinology and Metabolism, Kanazawa University Graduate School of Advanced Preventive Medical Sciences, Ishikawa 920-8640, Japan; Department of Endocrinology and Metabolism, Kanazawa University Graduate School of Medical Sciences, Ishikawa 920-8640, Japan; Department of Endocrinology and Metabolism, Kanazawa University Graduate School of Advanced Preventive Medical Sciences, Ishikawa 920-8640, Japan; Department of Endocrinology and Metabolism, Kanazawa University Graduate School of Medical Sciences, Ishikawa 920-8640, Japan; Department of Endocrinology and Metabolism, Kanazawa University Graduate School of Advanced Preventive Medical Sciences, Ishikawa 920-8640, Japan; Department of Bone and Joint Disease, National Center for Geriatrics and Gerontology, Obu, Aichi 474-8511, Japan; Department of Endocrinology and Metabolism, Kanazawa University Graduate School of Medical Sciences, Ishikawa 920-8640, Japan; Division of Basic Science and Translational Research, Kanazawa University, Kanazawa 920-8640, Japan; Division of Basic Science and Translational Research, Kanazawa University, Kanazawa 920-8640, Japan; Department of Medical Neuroscience, Kanazawa University Graduate School of Medical Sciences, Ishikawa 920-8640, Japan; Department of Medical Neuroscience, Kanazawa University Graduate School of Medical Sciences, Ishikawa 920-8640, Japan; Department of Public Health, Graduate School of Advanced Preventive Medical Sciences, Kanazawa University, Ishikawa 920-8640, Japan; Department of Public Health, Graduate School of Advanced Preventive Medical Sciences, Kanazawa University, Ishikawa 920-8640, Japan; Department of Public Health, Graduate School of Advanced Preventive Medical Sciences, Kanazawa University, Ishikawa 920-8640, Japan; Department of Endocrinology and Metabolism, Kanazawa University Graduate School of Advanced Preventive Medical Sciences, Ishikawa 920-8640, Japan; Department of Endocrinology and Metabolism, Kanazawa University Graduate School of Medical Sciences, Ishikawa 920-8640, Japan

**Keywords:** anxiety, diabetes type 2, metabolic stress, diet effects, HFHSD, selenoprotein P

## Abstract

**Context:**

Diabetes-associated metabolic stress and anxiety reciprocally influence one another's onset and course. We previously linked excessive selenoprotein P (SeP, encoded by *SELENOP* in humans) to pathological conditions frequently observed in individuals with diabetes.

**Objective:**

The present study aimed to clarify the role of SeP in the metabolic stress-induced anxiety.

**Methods:**

We visualized *Selenop* expression in the mouse brain section via RNAscope *in situ* hybridization and used RT-qPCR to evaluate gene expression in brain regions. We created brain-specific *Selenop* knockout (b*Selenop^-/-^*) mice by mating *Selenop*-flox and *Nestin*-Cre mice and conducted behavior tests for anxiety-like behavior and spatial memory under both a standard (STD) and high-fat, high-sucrose diet (HFHSD) conditions. In a cross-sectional general population cohort study, we examined differences in serum selenoprotein P concentrations between individuals with and without anxiety symptoms.

**Results:**

RNAscope *in situ* hybridization identified glial and endothelial cells as the sources of SeP synthesis in the brain. *Selenop* was expressed at the same level in the brains of mice fed with an STD and HFHSD. b*Selenop^-/-^* mice did not exhibit altered body weight or glucose tolerance associated with HFHSD feeding. High-fat, high-sucrose diet aggravated the anxiety-like behavior in the control mice, whereas *Selenop* deletion in the brain ameliorated the anxiety-like behavior without affecting spatial memory. Epidemiological data revealed that serum selenoprotein P was significantly higher in subjects with anxiety symptoms.

**Conclusion/interpretation:**

These findings suggest that excess SeP production may be a common trait linking metabolic stress with anxiety.

It has been recognized that metabolic stress in diabetes and obesity is associated with anxiety, which is exacerbated by behavioral, social, and biological factors (1, 2). Anxiety increases the risk of diabetes through stress-coping eating habits, low adherence to medication and self-care routines, increased cortisol and insulin resistance, and elevated inflammation. Conversely, diabetes elevates the risk of anxiety by psychological stress through diet and lifestyle intervention, lack of social support, and mood swings from glycemic fluctuations (3). Although the behavioral and social aspects of this relationship have been studied, the underlying molecular mechanisms remain largely unexplored.

Selenoprotein P (SeP, encoded by *SELENOP* in humans) is a hepatokine that acts as an antioxidant protein through its enzymatic action or by promoting the synthesis of other selenoproteins by supplying selenium (Se) to various cells (4, 5). We reported that SeP is upregulated in the diabetic liver and contributes to various pathological conditions commonly observed in diabetes, such as insulin resistance, impaired vasculogenesis, exercise resistance, and impaired thermogenesis (6-9). The glia produces SeP and delivers it to neurons via the ApoER2 receptor in the brain (10). SeP supports normal central nervous system (CNS) function by sustaining Se levels (11, 12) to prevent ataxia, dystonia, and neurodegeneration (13-17). Deletion of SeP in mice results in a psychiatric phenotype: impaired fear learning and sensorimotor gating because of poor parvalbumin neuron survival (18). Furthermore, low SeP levels disrupt spatial memory by deteriorating synaptic function in the hippocampus (19). Conversely, high SeP levels in blood and cerebrospinal fluid (CSF) are positively associated with an increased risk for the development of dementia in humans (20).

Based on these findings, we hypothesized that the overproduction of SeP in type 2 diabetes is related to the anxiety phenotype. In the present study, we first identified the localization of *Selenop* expression in the CNS using quantitative in situ hybridization (ISH) combined with immunohistochemical staining in various brain regions in mice fed a standard diet (STD) and a high-fat, high-sucrose diet (HFHSD). We created brain-specific *Selenop* KO (b*Selenop^-/-^*) mice by mating *Selenop*-flox and *Nestin*-Cre mice to determine the effect of SeP on anxiety behavior and spatial memory. Furthermore, we examined the relationship between serum full-length SeP (FL-SeP) levels and anxiety-related symptoms using data from an epidemiological study of the general population as human evidence.

## Materials and methods

### Animals

C57BL/6J male mice were used for this study. Brain-specific *Selenop* knockout (b*Selenop*^-/-^) mice were created by crossing *Selenop*-floxed (*Selenop*fl/fl) mice (9) with B6.Cg-Tg(Nes-cre)1Nogu (*Nestin-*Cre) mice (21), obtained from Vanderbilt University (United States) and RIKEN BRC through the National BioResource Project of the MEXT (Japan), respectively. *Selenop*-floxed littermates without Cre transgene were used as controls (*Selenop*^fl/fl^).

All animals were housed as groups in a 12-hour light/dark cycle and allowed free access to food and water. Mice were fed a STD (Oriental Yeast) and a rodent food containing 60% fat with an increased sucrose diet (HFHSD, D03062301, Research Diet).

The animal studies were performed according to the Guidelines on the Care and Use of Laboratory Animals issued by Kanazawa University and the ethics committee approved the protocol (approval no. AP23-031). All efforts were made to minimize animal suffering and to reduce the number of animals used.

### *In situ* hybridization

RNA scope ISH was performed on brain samples from *Selenop*^fl/fl^ and b*Selenop*^-/-^ mice fed a STD, *Selenop*^fl/fl^ and b*Selenop*^-/-^ mice fed a HFHSD. *In situ* hybridization was performed as described previously (22). The mice were deeply anesthetized and transcardially perfused with 4% paraformaldehyde (PFA) in PBS. The dissected brains were postfixed overnight with 4% PFA in PBS. The brains were cryoprotected by immersion in 30% sucrose in PBS for 3 days and embedded in an optimal cutting temperature compound. Coronal sections of 8-µm thickness were prepared using a cryostat. RNAscope ISH was done according to the manufacturer's instructions using the RNAscope 2.5 HD detection reagent kit RED (Advanced Cell Diagnostics, #322330). The sections were analyzed by immunohistochemistry. The mouse *Selenop* probe (#549611) was purchased from Advanced Cell Diagnostics. The *dapB* probe targeting the bacterial *dapB* gene was used as a negative control.

### Immunohistochemistry (following RNAscope ISH)

After RNAscope ISH, sections were permeabilized with 0.3% Triton X-100 in PBS and treated with a blocking solution (Blocking one histo, Nacalai Tesque, #L2E9350) containing 0.3% Triton X-100 in PBS. The sections were incubated overnight at 4 **°**C with anti-GS (Abcam, Cat# ab228590, rabbit, 1:500, RRID: AB_3665798), anti-Iba1 (Wako, Cat# 019-19741, RRID: AB_839504, rabbit, 1:1000), or NeuN (Cell Signaling Technology Cat# 12943, RRID: AB_2630395) primary antibodies and washed in PBS. After incubation with Alexa Fluor 488 (Invitrogen, anti-rabbit, Cat# A21206, RRID: AB_2535792) for 1 hour at room temperature, the sections were washed and mounted with a mounting medium containing DAPI (Vectashield Vibrance, Vector Laboratories, Cat# ZJ0926).

### Quantitation of *Selenop* signal

Coronal sections in the range between Bregma −1.65 to −2.05 were subjected to RNAscope ISH, GS immunohistochemistry, and DAPI staining. Images were obtained with a BZ-X710 Keyence microscope and analyzed using Fiji software. Astrocytes in the amygdala zone were randomly selected, and the number of *Selenop* puncta in the cytoplasm of GS-positive cells was counted. Mean fluorescence intensity was automatically calculated using Fiji software.

### Behavior experiments

Behavior tests were conducted in the following order: elevated plus maze (EPM) and open field (OF) tests at 12-13 weeks of age with a 1-day break between tests, and the Barnes maze (BM) was performed at 18-20 weeks of age on an STD. After 2 months of HFHSD, the mice were tested for EPM and OF at 29-30 weeks of age and for BM at 35-37 weeks of age.

Experiments were performed during the light phase. Seven *Selenop*^fl/fl^ mice and 9 b*Selenop*^-/-^ mice were evaluated. The mice were housed in groups of 3-5 per cage. After each test, a damp towel and 1% sodium hypochlorite, followed by 70% alcohol, were used to clean feces and remove odor. All tests were recorded with a video system placed above the equipment and done in the same experimental room. Mice in the home cages were placed in the experimental room 30 minutes before the test. After the individual test, a mouse was placed in a temporary clean cage. At the end of the entire test, all mice were placed into the home cage. The experiments were performed on different days, with at least 1 day of rest between tests. The behavior experiments were analyzed using a digital video system and ANY-maze video tracking software (Stoelting Co., Wood Dale, IL, United States).

#### Elevated plus maze

Elevated plus maze testing was performed as described previously (23). The maze apparatus was elevated to 50 cm above the floor and consisted of a central platform (5 × 5 cm) from which 2 open arms (5 × 25 cm without walls) and 2 closed arms (5 × 25 cm with 15-cm-high transparent walls) were extended in opposite directions. Mice were placed onto the central platform facing an open arm and were allowed to move freely for 5 minutes. The total distance and time spent in the closed and open arms were calculated.

#### Open field

Open field was performed as described previously (24). Mice were placed in the center of the OF chamber and allowed to move freely for 10 minutes. The OF chamber was a square wooden box (60 × 60 × 20 cm) covered inside with polypropylene sheets. The OF was divided into an inner zone (30 × 30 cm) and the periphery. The total distance and time spent in the inner and outer zones of the arena were calculated.

#### Barnes maze

The test was performed as described previously (25, 26). Mice were placed in the center of an elevated (80 cm above the floor) circular platform (91 cm in diameter) containing 20 holes around the periphery. The holes had a uniform diameter of 5 cm and appearance; however, only 1 hole was connected to a removable black escape box (20 × 10 × 9 cm). A starting box with a circular platform (20 cm in diameter) and a lid was used to place the mouse onto the platform. Four visual cues were placed on each of the 4 sides of the BM with different shapes and colors. The apparatus was surrounded by white screens to avoid distracting environmental cues from the room. The experiments were performed in the light phase from 9 Am to 4 Pm. The light intensity was set at 1000 lux, which induced mild aversive stimuli to motivate the mice to search for shelter in the escape box. A video camera mounted in the center above the circular arena recorded all of the movements of the mice. The time from the start of the trial to the moment when the mouse entered the escape box was defined as the latency to reach the escape hole and was analyzed using Animaze software. The number of holes explored before the mouse discovered the escape hole was considered a primary error and calculated manually.

The test consisted of 3 stages: habituation, acquisition, and probe day. Habituation took place on the first day and consisted of placing the animal in the escape box for 3 minutes and in the arena for 1 minute. Immediately afterward, the first training of the acquisition stage occurred: the animal was placed by the experimenter in the middle of the platform covered by a starting box for 20 seconds and then released to find the escape hole. If the mouse failed to find the escape box within 2 minutes, it was gently guided to the box and remained there for 1 minute before returning to the home cage. The experiment continued for 4 consecutive days with 4 trials each day at 30-minute intervals. On the fifth day, the mice were subjected to a probe test. The escape box was removed, and the animals could move freely for 2 minutes. Only 1 trial was conducted per animal.

### Glucose tolerance test

A glucose tolerance test (GTT) was performed at 20 weeks of age on mice fed an STD and at 38 weeks of age on an HFHSD as described previously (27). Body weight was measured a day before testing. Briefly, the mice were fasted for 12 hours and received intraperitoneal glucose injections at 1.5 g/kg for the STD group and 0.375 g/kg for the HFHSD group. The HFHSD group received one-quarter of the glucose dose administered to the STD group in order to prevent excessive hyperglycemia and ensure glucose levels remained within a measurable range. Blood glucose levels were measured before and 15, 30, 60, 90, and 120 minutes following injection.

### Quantitative RT-PCR

Following behavior tests and GTT, aged animals were dissected for gene analysis. An additional STD group was tested at the same age as the initial behavioral testing. The mice were anesthetized and dissected to collect the liver and brain regions: cortex, hippocampus, cerebellum, hypothalamus, and olfactory bulb. To preserve RNA integrity, tissues were isolated on an ice-cold metal block, stabilized in RNA later for 24 hours, frozen in liquid nitrogen, and stored at −80 °C. Total RNA was extracted from the mouse brain regions and liver using the RNeasy Lipid Tissue Mini RNA Isolation Kit (QIAGEN). The RNA (100 ng) was reverse-transcribed using a high-capacity cDNA reverse transcription kit (Thermo Fisher Scientific). The quality of total RNA was confirmed by ribosomal RNA degradation. Quantitative polymerase chain reaction (qPCR) was done using Applied Biosystems TaqMan probes (Sepp1, Mm00486048_m1, NCBI RefSeq NM_001042613.1, NM_001042614.1; Gpx1, Mm00656767_g1, NCBI RefSeq: NM_008160.6; Gapdh, 4351309, NCBI RefSeq: NM_008084; and the StepOnePlus real-time PCR system (Applied Biosystems by Life Technologies). The procedures were performed according to the manufacturer's instructions.

### Epidemiological studies of the general population

We obtained data from a cross-sectional analysis called the SHIKA-Study, conducted in Shika Town, Ishikawa Prefecture, from 2013 to 2019, to monitor the health status of residents and explore preventive measures for lifestyle-related diseases (28).

A total of 1335 adults aged 40 years or older from the study area voluntarily participated in health examinations, answered health questionnaires, and provided blood samples. Of these, 187 participants completed a single screening item assessing restlessness (ie, “feeling so restless that it is hard to sit still”) on a 5-point frequency scale (0-4), a symptom included in DSM-5 criteria for generalized anxiety disorder and in the GAD-7 questionnaire (29, 30), and underwent serum FL-SeP measurements. Because responses were sparse and the distribution was skewed, we dichotomized the item (present vs absent) for regression analyses to improve estimation stability and statistical power.

Serum FL-SeP, age, BMI, and HbA1c were compared between participants, 99 males and 88 females, with and without anxiety symptoms using the Mann–Whitney *U* test. Multivariate *P*-values were obtained from binary logistic regression analyses.

This epidemiological study was approved by the Kanazawa University Hospital Human Research Ethics Committee (1491, 2016-376) and conducted in accordance with the principles of the Declaration of Helsinki. Written informed consent was obtained from all participants.

### Statistical analysis

Statistical analysis was performed using Prism 8 software (GraphPad Software Inc., San Diego, CA, United States). Sample sizes, statistical tests, and statistical significance are described in the Figure legends and Results. All *n* values indicate the number of animals.

## Results

### Glial *Selenop* expression in the anxiety-associated amygdala

**Figure 1:**
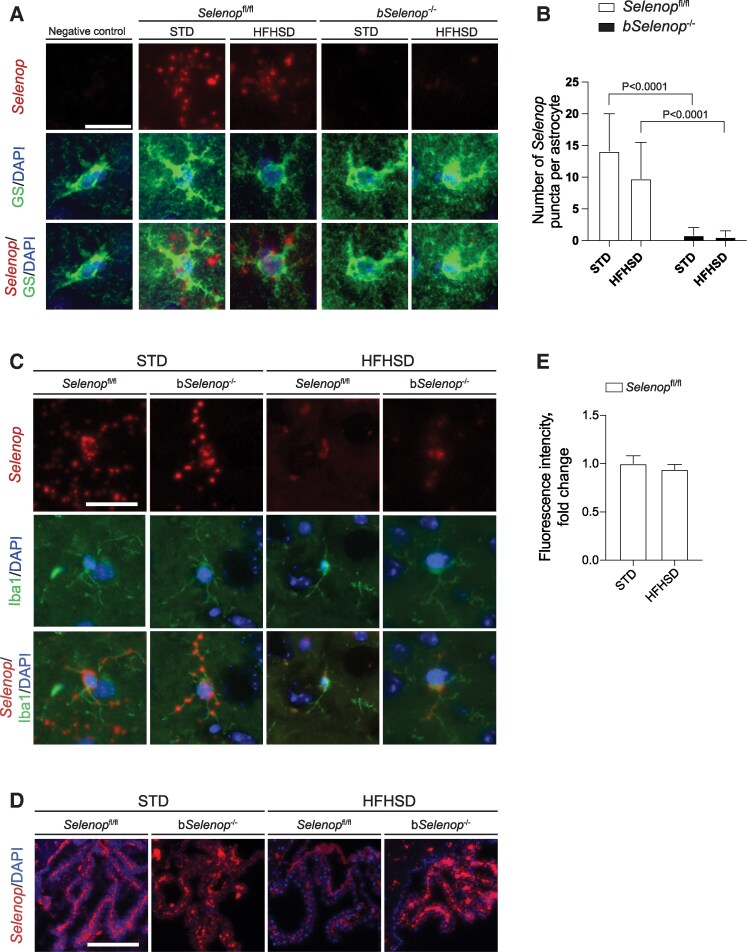
*Selenop* expression in the amygdala. RNAscope ISH was performed in *Selenop*  ^fl/fl^ mice and b*Selenop*^-/-^ mice in the STD and the HFHSD groups. (A-B) RNAscope ISH of coronal sections of the amygdala for *Selenop* and immunostaining with anti-GS antibody (A) and the number of *Selenop* puncta per astrocyte (B), (C) RNAscope ISH of coronal sections of the amygdala for *Selenop* and immunostaining with anti-Iba1 antibody, (D) RNAscope ISH of coronal sections of the choroid plexus for *Selenop* without immunostaining, (E) Quantification of the fluorescence intensity of the *Selenop* signal in the amygdala regions of *Selenop*^fl/fl^ mice. In (B, E), the data represent the mean ± SD; *n* = 3 mice for each condition; white boxes = control, black boxes = b*Selenop*^-/-^. STD, standard diet, HFHSD, high-fat high-sucrose diet. Statistical significance between different diets in the same genotype and different genotypes in the same diet was calculated using a 2-tailed unpaired Mann–Whitney test, significant *P*-values are indicated as exact numbers. Scale bars (A, C) 20 µm, (D) 150 µm. Scale bars (A, C) 20 µm, (D) 150 µm.

To visualize *Selenop* expression and confirm the source of SeP in the brain of *Selenop*^fl/fl^ mice, we performed RNAscope ISH along with immunostaining using antibodies against glutamine synthetase (GS) as an astrocyte marker (Fig. 1A and 1B), ionized calcium-binding adaptor molecule 1 (Iba1) as a microglia marker (Fig. 1C), and neuronal nuclear protein (NeuN) as a neuron marker. The results indicated that *Selenop* expression in the brain was present in astrocytes (Fig. 1A) and microglia (Fig. 1C) of the stress and fear-related amygdala region and the endothelium of the choroid plexus (Fig. 1D). *Selenop* expression was absent in the neurons (Fig. S1A (31)). These results are consistent with previous findings by RNA sequencing of the cortex showing that *Selenop* expression is prevalent in non-neuronal cells of the brain, such as the glia and endothelium (32). Due to glial origins, *Selenop* is detected across the brain (Fig. S1B (31)), consistent with the Allen brain atlas (33).

### HFHSD did not affect brain *Selenop* expression

**Figure 2:**
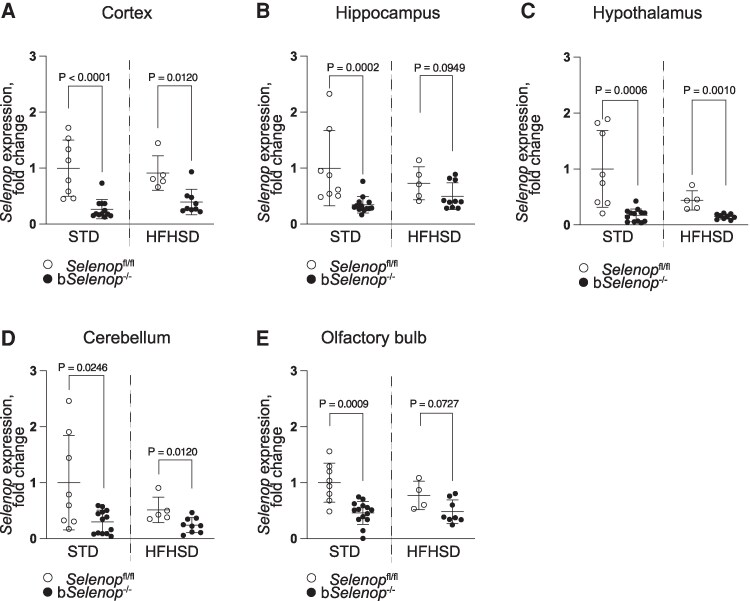
*Selenop* expression in various brain regions. RT-PCR was performed in *Selenop*  ^fl/fl^ mice and b*Selenop*^-/-^ mice in the STD and the HFHSD groups. (A-E) *Selenop* mRNA expression in the (A) hippocampus, (B) cortex, (C) hypothalamus, (D) cerebellum, and (E) olfactory bulb of the brain. Gene expression was measured by real-time PCR. Expression values were normalized to that of *Gapdh* mRNA. *n* = 8 for *Selenop*^fl/fl^ on STD; *n* = 13 for b*Selenop*^-/-^on STD, *n* = 5 for *Selenop*^fl/fl^ on HFHSD; *n* = 10 for b*Selenop*^-/-^ on HFHSD. Dots are individual values, and error bars are SD; white circles = *Selenop*^fl/fl^; black circles = b*Selenop*^-/-^; STD, standard diet, HFHSD, high-fat high-sucrose diet; Statistical significance between different diets in the same genotype and different genotypes in the same diet were calculated using 2-tailed unpaired Mann–Whitney test, significant *P*-values are indicated as exact numbers.

To identify diet-induced changes in *Selenop* expression, we measured the signal intensity of slides stained by RNAscope ISH (Fig. 1E) and performed RT-qPCR (Fig. 2) of brain samples from mice fed an STD or an HFHSD. The HFHSD significantly upregulated *Selenop* expression in the liver (Fig. S2 (31)) but did not affect gene expression in the brains of *Selenop*^fl/fl^ mice (Fig. 1E, 2A-2E).

### *Nestin*-Cre mediated deletion of *Selenop* in the brain

To determine the effect of SeP in the brain, we generated b*Selenop^-/-^* mice by crossing *Nestin*-Cre with *Selenop*-floxed mice and used *Selenop*-flox littermates as controls (*Selenop*^fl/fl^). It was previously shown that *Nestin*-Cre recombination is observed in astrocytes, but not in the microglia or endothelium (21). We performed RNA scope ISH in different regions of the brains of b*Selenop*^-/-^ mice. Astrocytes in the amygdala region of b*Selenop*^-/-^ mice expressed significantly lower levels of *Selenop* mRNA on an STD or HFHSD (Fig. 1A and 1B). The cortex (Fig. 2A), hippocampus (Fig. 2B), cerebellum (Fig. 2C), hypothalamus (Fig. 2D), and olfactory bulb (Fig. 2E) also exhibited significantly reduced *Selenop* expression in the b*Selenop*^-/-^ groups.

### Brain-specific *Selenop* deletion did not affect body weight and glucose tolerance

**Figure 3:**
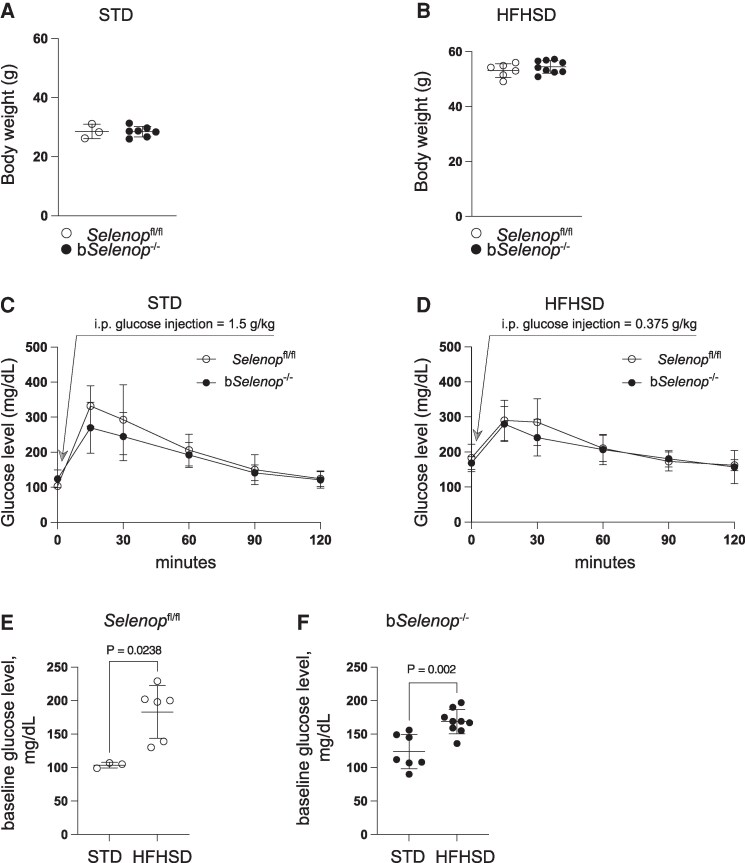
Effect of brain-specific *Selenop* KO on body weight and glucose tolerance. (A) Body weight of *Selenop*^fl/fl^ (*n* = 3) and b*Selenop*^-/-^ (*n* = 7) on STD. (B) Body weight of *Selenop*^fl/fl^ (*n* = 6) and b*Selenop*^-/-^ (*n* = 9) on HFHSD. (C) Glucose tolerance of *Selenop*^fl/fl^ (*n* = 3) and b*Selenop*^-/-^ (*n* = 7) on STD. (D) Glucose tolerance of *Selenop*^fl/fl^ (*n* = 6) and b*Selenop*^-/-^ (*n* = 9) on HFHSD. (E) Baseline glucose level of *Selenop*^fl/fl^ on STD (*n* = 3) and HFHSD (*n* = 6). (F) Baseline glucose level of b*Selenop*^-/-^ on STD (*n* = 7) and HFHSD (*n* = 9). Mice fed STD and HFHSD received intraperitoneal glucose injections of 0.375 and 1.5 g/kg, respectively. In (A-B) and (E-F) dots are individual values and error bars are SD; in (C) and (D), dots represent mean values ± SD, white circles = *Selenop*^fl/fl^, black circles = b*Selenop*^-/-^. STD, standard diet, HFHSD, high-fat high-sucrose diet. Statistical significance was calculated by a 2-tailed unpaired Mann–Whitney test (A-B, E-F), and by area under the curve (AUC) analysis (C-D). There was no statistical difference in body weight (A, B) and glucose tolerance (C, D) between genotypes. Significant *P*-values are indicated as exact numbers (E-F).

To assess some key systemic metabolic parameters of brain-specific *Selenop* KO, we measured the body weight, hepatic expression of *Selenop*, and performed a GTT (1.5 g/kg for mice fed STD and 0.375 g/kg for mice fed HFHSD) (Fig. 3). The HFHSD feeding increased body weight (Fig. 3A and 3B), showed similar glucose excursions even at ¼ of glucose load (Fig. 3C and 3D), elevated fasting baseline glucose levels (Fig. 3E and 3F), and upregulated hepatic expression of *Selenop* (Fig. S2 (31)) with no significant differences between the genotypes. These results suggest that brain-specific *Selenop* deletion does not affect body weight, glucose tolerance, or liver *Selenop* expression in the mice.

### *Selenop* deletion mitigated anxiety-like behavior

**Figure 4:**
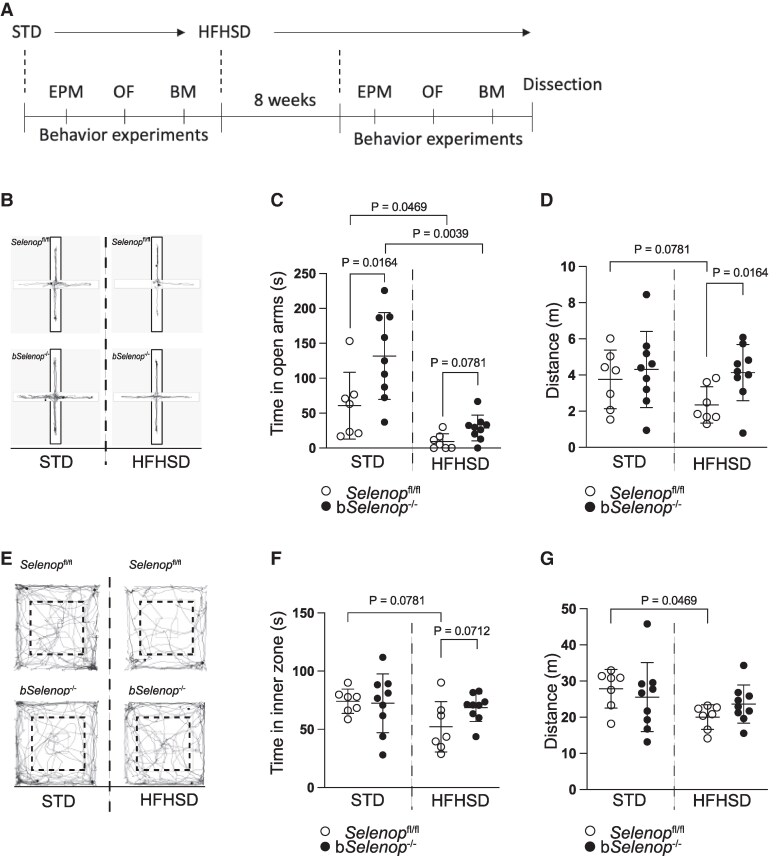
Anxiety-like behavior testing in the elevated plus maze (EPM) and open field (OF) tests. EPM and OF were performed in *Selenop*  ^fl/fl^ mice and b*Selenop*^-/-^ mice fed an STD and retested after 8 weeks of HFHSD feeding. (A) Timeline for anxiety-like behavior testing: *Selenop*^fl/fl^ and b*Selenop*^-/-^ mice were maintained on an STD for baseline assessment of anxiety-like behavior, followed by 8 weeks of an HFHSD feeding, after which behavioral testing was repeated. (B) Representative track plots of EPM, (C) time in the open arms, (D) distance traveled in the arena by *Selenop*^fl/fl^ and b*Selenop*^-/-^ mice in the EPM test before and after HFHSD feeding. (E) Representative track plots for OF, (F) time in the inner zone, (G) distance traveled by mice in the OF before and after HFHSD feeding. (*n* = 7 for *Selenop*^fl/fl^ mice, *n* = 11 for b*Selenop*^-/-^ mice). Dots represent individual values, and error bars are SD; white circles = *Selenop*^fl/fl^; black circles = b*Selenop*^-/-^; STD, standard diet, HFHSD, high-fat high-sucrose diet. Statistical significance between different diets in the same genotype was calculated using a 2-tailed Wilcoxon matched-pairs signed rank test. Statistical significance between different genotypes in the same diet was calculated using a 2-tailed unpaired Mann–Whitney test, significant *P*-values are indicated as exact numbers.

To test anxiety-like behavior in the b*Selenop*^-/-^ mice, an EPM and OF tests were performed. Mice feel more comfortable in enclosed spaces; thus, less time in the open arms of the EPM and in the inner zone of the OF arena indicates anxiety-like behavior. We repeated the behavior tests under STD feeding and 8 weeks of HFHSD feeding in the same mouse (Fig. 4A).

In the EPM test (Fig. 4B-4D), the b*Selenop*^-/-^mice spent significantly more time in the open arms compared with the *Selenop*^fl/fl^ mice on an STD or HFHSD (Fig. 4C). After 2 months of an HFHSD, time in the open arms significantly decreased for both genotypes (Fig. 4C). *Selenop*^fl/fl^ mice tended to walk less after HFHSD feeding, whereas b*Selenop*^-/-^ mice maintained their levels of locomotor activity (Fig. 4D). In the OF test (Fig. 4E-4G), HFHSD significantly reduced the time spent in the inner zone for the *Selenop*^fl/fl^ mice, but not the b*Selenop*^-/-^ mice (Fig. 4F). Similarly, an HFHSD reduced the walking distance in the *Selenop*^fl/fl^ mice, but not in the b*Selenop*^-/-^ mice (Fig. 4G). Taken together, these data suggest that *Selenop* deletion in the brain partly contributes to the moderation of HFHSD-induced anxiety-like behavior.

### *Selenop* deletion does not affect spatial memory

**Figure 5:**
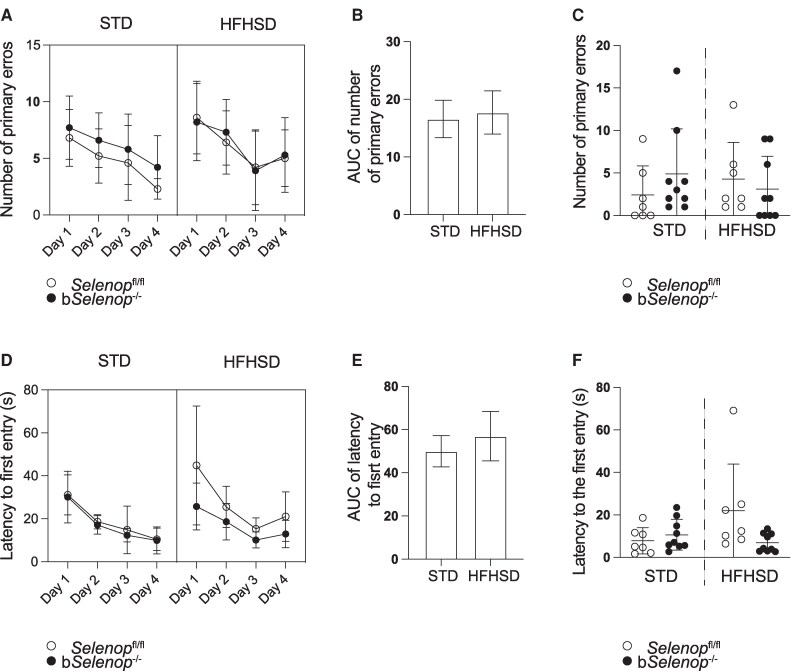
Spatial memory assessment using the Barnes Maze (BM). BM was performed in *Selenop*  ^fl/fl^ mice and b*Selenop*^-/-^ mice in the STD and retested after 8 weeks of HFHSD feeding. A) Number of errors during acquisition. (B) The area under the curve (AUC) analysis for the number of primary errors in each diet group in (A). The number of errors of *Selenop*^fl/fl^ and b*Selenop*^-/-^ mice in each diet group was combined to calculate the AUC. (C) Number of errors on the test day. (D) Latency to the first entry to the escape hole during acquisition in *Selenop*  ^fl/fl^ mice and b*Selenop*^-/-^ mice in the STD and the HFHSD groups. (E) The AUC analysis for the primary latency in each diet group in (D). The number of errors of *Selenop*^fl/fl^ and b*Selenop*^-/-^ mice in each diet group was combined to calculate the AUC. (F) Latency to reach the zone where the escape hole used to be on the test day. *N* = 7 for *Selenop*^fl/fl^ mice, *n* = 11 for b*Selenop*^-/-^ mice. In (A, B, D, E), dots and bars represent the mean ± SD; in (C) and (F), dots are individual values, and error bars are SD; white circles = *Selenop*^fl/fl^; black circles = b*Selenop*^-/-^; STD, standard diet, HFHSD, high-fat high-sucrose diet. Statistical significance between different diets in the same genotype was calculated using 2-tailed Wilcoxon matched-pairs signed rank test. Statistical significance between different genotypes in the same diet was calculated using a 2-tailed unpaired Mann–Whitney test. Significant *P*-values are indicated as exact numbers.

Given that low SeP levels have been reported to impair spatial memory, we also assessed the spatial memory phenotype in b*Selenop*^-/-^ mice. To test spatial memory, we conducted a BM test. The mice were trained 4 times a day for 4 days to memorize the position of the escape hole. The average number of errors in locating the escape hole (Fig. 5A) and latency for the first entry to the hole (Fig. 5C) indicated that the *Selenop*^fl/fl^ and b*Selenop*^-/-^ mice successfully memorized the escape path in the maze within 4 days. There was no significant difference in the pace of acquisition between groups (Fig. 5A and 5D) or diets (Fig. 5B and 5E). On the 5th day, the escape hole was removed to confirm that the mice had learned the escape route. Both *Selenop*^fl/fl^ and b*Selenop*^-/-^ groups passed the test. There was no difference in the number of primary errors (Fig. 5C) or latency for the first entry (Fig. 5D) between groups and diet conditions. These results suggest that an HFHSD and *Selenop* deletion do not affect spatial memory.

### *Selenop* deletion does not affect *Gpx1* expression in the brain

**Figure 6:**
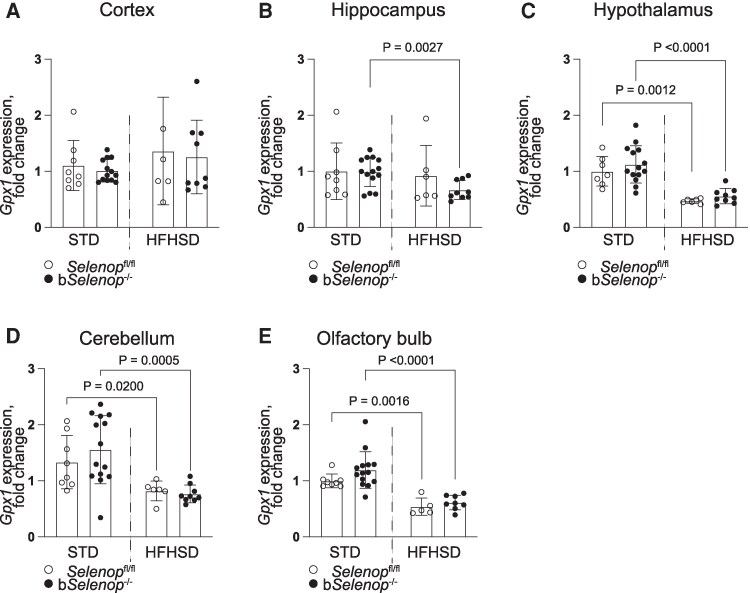
Gpx1 expression in various brain regions. RT-PCR was performed in *Selenop*  ^fl/fl^ mice and b*Selenop*^-/-^ mice in the STD and the HFHSD groups. The mRNA expression of Gpx1 in the (A) hippocampus, (B) cortex, (C) hypothalamus, (D) cerebellum, and (E) olfactory bulb of the mouse brain. Each gene expression was assessed by real-time PCR. Expression values were normalized to *Gapdh* mRNA. *n* = 8 for *Selenop*^fl/fl^ on STD; *n* = 10 for b*Selenop*^-/-^ on STD, *n* = 6 for *Selenop*^fl/fl^ on HFHSD; *n* = 9 for b*Selenop*^-/-^ on HFHSD. Dots are individual values and error bars are SD; white circles = *Selenop*^fl/fl^; black circles = b*Selenop*^-/-^; STD, standard diet; HFHSD, high-fat high-sucrose diet; Gpx1 encodes glutathione peroxidase 1; Statistical significance between different diets in the same genotype and different genotypes in the same diet was calculated using 2-tailed unpaired Mann–Whitney test, significant *P*-values are indicated as exact numbers.

To elucidate the potential mechanism of *Selenop*-associated anxiety-like behavior, we measured the expression of selenium-dependent antioxidant glutathione peroxidase 1 (*Gpx1)* in the brain by RT-PCR (Fig. 6). The HFHSD significantly downregulated *Gpx1* expression in the hypothalamus (Fig. 6C), cerebellum (Fig. 6D), and olfactory bulb (Fig. 6E) in both the *Selenop*^fl/fl^ and b*Selenop*^-/-^ groups, and in the cortex (Fig. 6A) of the b*Selenop*^-/-^ mice. In contrast, the expression of hippocampal *Gpx1* did not depend on diet (Fig. 6B). *Selenop* deletion did not affect *Gpx1* expression in any of these regions.

### Human epidemiological evidence for the association between SeP levels and anxiety symptoms

In an exploratory cross-sectional analysis, serum FL-SeP concentrations were significantly higher in participants reporting restlessness-related symptoms (*P* = .026). Restlessness is one of the DSM-5–listed associated symptoms of generalized anxiety disorder.

After adjusting for confounding factors such as age, sex, BMI, and HbA1c levels, the association between serum FL-SeP concentrations and the presence of restlessness-related symptoms remained significant (Table 1).

**Table 1:** Univariate and multivariate regression analysis of the association between Serum FL-SeP and other risk factors and restlessness-related symptoms.

Predictor	Univariate	Multivariate	OR (95% CI)
	*P* value	*P* value	
Serum FL-SeP (mg/L)	.026*^a^*	.038*^a^*	1.71 (1.03-2.82)
Sex (male)	.59	.048*^a^*	2.03 (1.01-4.08)
Age (year)	<.001*^a^*	.017*^a^*	0.96 (0.93-0.99)
BMI (kg/m^2^)	.037*^a^*	.038*^a^*	0.89 (0.80-0.99)
HbA1c (%)	.96	.17	1.62 (0.82-3.22)

Relationship between serum full-length selenoprotein P (FL-SeP) concentrations and the presence or absence of restlessness, a core symptom of generalized anxiety disorder. Serum FL-SeP concentrations were compared between individuals with (*n* = 56) and those without (*n* = 131) restlessness symptoms. Univariate *P*-values were obtained from the χ^2^ test for sex and Mann–Whitney *U* test for Serum FL-SeP, age, BMI, and HbA1c. Multivariate *P*-values were from binary logistic regression analyses.

^*a*^Significant difference (*P* < .05).

## Discussion

In this study, we investigated the role of SeP in anxiety-like behavior under dietary metabolic stress using b*Selenop*^-/-^ mice. We also analyzed epidemiological data in humans to examine the relationship between serum FL-SeP levels and anxiety symptoms. These findings suggest that SeP may act as a mediator linking metabolic abnormalities and anxiety-related behavior, supported by consistent evidence from both mouse and human models.

First, we demonstrated that *Selenop* is strongly expressed in glia within the brain (Fig. 1 and Fig. S1 (31)). This complements the conventional understanding of SeP as a liver-derived selenium carrier and suggests an intrinsic functional role within the CNS.

*Nestin*-mediated *Selenop* KO reduced *Selenop* expression in astrocytes, whereas it did not affect the expression in the microglia and endothelium (Fig. 1). Therefore, any behavioral alterations are considered as the result of impaired SeP production from the astrocytes, which emphasizes the previously recognized role of astrocytes in anxiety modulation (34, 35). These results underscore that astrocytic SeP, rather than global brain levels, drives anxiety alterations in anxiety behaviors in our model.

In behavioral experiments, control *Selenop*^fl/fl^ mice increased markedly in anxiety-like behaviors following HFHSD feeding. In contrast, such changes were significantly attenuated in b*Selenop*^-/-^ mice (Fig. 4D and 4G). These results imply that brain-derived SeP contributes to the manifestation of anxiety behavior under metabolic stress. Despite the clear anxiety phenotype, spatial memory as assessed by the BM remained intact after astrocytic SeP deletion (Fig. 5). These results differ from the previous study using systemic *Selenop* knockout mice that show memory deficits in the Morris Water Maze (19).There may be 2 reasons for these results. First, the Morris water maze may be a more sensitive test than the BM because of the longer training period (9 days vs 4 days). Second, microglial and endothelial cells may compensate for a shortage of SeP synthesis in astrocytes to maintain spatial memory function. Future studies employing longer training paradigms or combined glial-targeted approaches could clarify SeP's role in learning and memory.

In human data, we also observed that individuals with higher serum FL-SeP levels had an increased risk of anxiety symptoms (Table 1). This association remained significant after adjusting for sex, age, BMI, and other covariates, indirectly suggesting that circulating SeP may influence CNS function. Although the liver is the primary source of SeP, some studies have reported correlations between serum and CSF SeP levels in neurological diseases (36), supporting the possibility that SeP serves as a marker or effector bridging metabolic states and mental health.

SeP may influence anxiety via reductive stress, a condition in which excessive antioxidant activity suppresses physiological levels of ROS required for normal intracellular signaling (37). To date, we do not know exactly the molecular target of SeP-mediated reductive stress until we obtain the redoxome landscape of the brain under anxiety conditions. However, one of the targets of SeP in the present study may be the vascular endothelial growth factor (VEGF) signaling. VEGF signaling plays a critical role in neurogenesis, synaptic plasticity, and emotional regulation (38, 39). A previous study reported that mice with elevated VEGF signaling exhibit reduced anxiety in behavioral tests. We previously showed that SeP causes VEGF resistance by eliminating ROS burst necessary for VEGF signaling, leading to impaired vasculogenesis in the endothelial cells (7, 40). Therefore, we hypothesize that SeP may scavenge the physiological ROS essential for VEGF signaling in the brain, thereby inhibiting neurogenesis and synaptic plasticity and contributing to anxiety phenotypes, which should be experimentally proved in the future.

This study has several limitations. First, CSF SeP levels were not directly measured in humans, leaving uncertainty about whether serum SeP accurately reflects central SeP function. Future studies should quantify SeP in CSF and investigate its transport pathways into the brain. Furthermore, anxiety was self-reported and not assessed by a full multi-item scale. Second, in the animal studies, only male mice were used. Future studies should include animals of both sexes. Third, the ages of the STD and the HFHSD groups are different at the time of the behavior experiments. However, this study aimed to longitudinally evaluate the effects of dietary change in the same individuals over time. After conducting behavioral tests under the STD condition, subjects were switched to the HFHSD and maintained for 8 weeks. Subsequently, behavioral tests and metabolic tests (GTT) were performed again under the HFHSD condition. Therefore, due to the experimental design, it was inevitable that the HFHSD group would be older than the STD group. Consequently, the age difference represents a “structural feature of the longitudinal model,” and the design prioritized “comparing changes before and after dietary intervention” over individual variation. Additionally, we are not confident that 8 weeks of HFHSD caused glucose intolerance at the time of the behavior testing. However, the HFHSD exaggerates the anxiety phenotypes as observed in Fig. 4B and 4C. These findings suggest that HFHSD itself caused metabolic stress on the anxiety phenotype, as observed in previous studies (41-43). Additionally, we confirmed the glucose intolerance after, but not before, the behavior testing in mice fed the HFHSD.

In conclusion, SeP, previously recognized as a diabetes-associated hepatokine, exhibited stable expression in the brain of mice fed an STD or HFHSD. *Nestin*-mediated deletion of *Selenop* in the astrocytes ameliorated the HFHSD-induced anxiety phenotype without affecting glucose metabolism, body weight, or spatial memory. While previous preclinical studies demonstrated that SeP-neutralizing antibodies can restore insulin sensitivity and improve β-cell function (44), our findings suggest a potential extension of this therapeutic strategy to metabolic-associated psychiatric conditions. Specifically, we show that targeting glial *Selenop* may have beneficial effects on anxiety, supporting the broader applicability of SeP-targeted interventions in diabetes-related neurobehavioral disorders.

## Data Availability

The data supporting the findings of this study are available from the corresponding author upon reasonable request.

## Abbreviations

BM: Barnes Maze
EPM: elevated plus maze
Gpx1: glutathione peroxidase 1
GS: glutamine synthetase
GTT: glucose tolerance test
HFHSD: high-fat high-sucrose diet
Iba1: allograft inflammatory factor 1 (ionized calcium-binding adapter molecule 1)
ISH: *in situ* hybridization
KO: knockout
OF: open field
PV: parvalbumin
SeP: Selenoprotein P
STD: standard diet
